# Study on Preparation of Zinc Pyrithione Ultrafine Particles via Rapid Precipitation in Microchannel Reactor

**DOI:** 10.3390/molecules31152621

**Published:** 2026-07-28

**Authors:** Guo-En Li, Yue Liu

**Affiliations:** 1 Department of Environmental and Chemical Engineering, Hebei Vocational University of Industry and Technology, Shijiazhuang 050091, China; liguoen1987@163.com; 2 Hebei Technology Innovation Center for Fine Chemicals Preparation in Green Process, Hebei Chemical & Pharmaceutical Vocational Technology College, Shijiazhuang 050026, China

**Keywords:** zinc pyrithione, microchannel reactor, rapid precipitation, ultrafine particles, process optimization

## Abstract

Aiming at the problems of large particle size (2–5 μm) and broad particle size distribution of zinc pyrithione (ZPT) particles prepared by traditional tank reactors, ultrafine ZPT particles were synthesized via rapid precipitation method in a microchannel reactor using sodium pyrithione (SPT) and zinc sulfate as raw materials. The effects of key parameters including reactant concentration, microchannel flow velocity and channel dimension on particle size and size distribution were systematically investigated by single-factor experiments, and the drying strategy was further optimized. The results showed that under the optimal conditions—SPT concentration of 0.5 mol/L, zinc sulfate concentration of 0.25 mol/L, channel flow velocity of 21.7 m/s, channel inner diameter of 0.8 mm, room temperature reaction and pressure of 1 MPa—the particle size of wet-state ZPT reached 180 nm, which was reduced by 40% compared with that prepared in tank reactors, and the corresponding polydispersity index (PDI) was 0.18. Spray drying was verified as the optimal drying method, by which the dried particles maintained a stable size of 600 nm with favorable dispersibility, and the Zeta potential was −20.6 mV. X-ray diffraction (XRD) and thermogravimetric analysis (TGA) characterizations confirmed that the as-prepared products possessed high purity and outstanding thermal stability with stable structure below 200 °C. This work provides an efficient technical route for the fabrication of narrow-dispersed ultrafine ZPT particles, and offers reliable experimental data for the industrial application of microchannel technology in ZPT synthesis.

## 1. Introduction

Zinc pyrithione (ZPT), with the molecular formula C_10_H_8_N_2_O_2_S_2_Zn and molecular weight of 317.7, is a faint white-yellow powdery solid with a melting point of 240 °C and a specific gravity of 1.782 at 25 °C. As a broad-spectrum bactericide, ZPT exhibits a distinctive antibacterial mechanism different from conventional bactericides. Instead of killing bacteria and fungi by releasing toxic substances, ZPT acts as a transmembrane carrier that transports extracellular protons into cells and expels intracellular potassium ions. Repeated ion exchange processes disrupt the transmembrane ion gradient, block intracellular nutrient transport and eventually lead to microbial death. Notably, ZPT is not consumed during the sterilization process and circulates repeatedly across cell membranes, thus avoiding the emergence of microbial drug resistance [1,2].

The practical application performance of ZPT is closely correlated with its particle size, size distribution and dispersion property. Nano-sized ZPT can be stably and uniformly suspended in carrier solvents. Smaller particle size endows ZPT with larger specific surface area, which facilitates its adhesion on antibacterial interfaces, prolongs antibacterial duration and reduces the dosage per unit area. In addition, nano-ZPT with narrow particle size distribution presents uniform and consistent bactericidal activity owing to superior particle homogeneity [3].

At present, four mainstream synthetic routes are adopted for laboratory research and industrial production of ZPT worldwide, which take 2-aminopyridine, pyridine-2-carboxylic acid, pyridine and 2-chloropyridine as starting materials, respectively [4]. The final precipitation step of all the above synthetic processes is performed in conventional tank reactors. In such reactors, two reactant solutions are mixed under long-term intense stirring to generate white ZPT precipitates, which belongs to typical complete mixing flow mode. Complete mixing flow reactors feature wide residence time distribution and severe backmixing behavior. The newly formed fine ZPT nuclei tend to grow on the surface of pre-existing large particles in the reactor, resulting in large particle size, uneven particle dimension and broad size distribution of final products [5,6].

Using microchannels to form uniform micro droplets for liquid–liquid interface reactions: preparing nanoparticles, MOFs materials, polymer microspheres, and liposomes. The droplet size is highly uniform, resulting in narrow particle size distribution of nano products. There are many research reports on the preparation of nanoscale particles such as barium sulfate and calcium carbonate using this method. However, there are few reports on the preparation of ZPT ultrafine particles using this method. To overcome the inherent defects of traditional complete mixing flow reactors, this study proposed a novel synthesis strategy for ZPT via liquid–liquid rapid precipitation in microchannel reactors. Microchannel reactors effectively eliminate backmixing phenomenon, enabling the preparation of ZPT particles with smaller size and larger specific surface area, and remarkably improving the comprehensive performance of final products [7,8,9]. In this study, the key reaction parameters for ZPT synthesis in liquid–liquid microchannel reactors were systematically optimized, aiming to explore a feasible microchannel synthesis strategy for high-quality ZPT and provide preliminary theoretical and experimental reference for its subsequent industrial optimization and application research. In this study, the one-factor-at-a-time (OFAT) method was applied to optimize operating conditions for the precipitation-crystallization process within microchannels. During the preliminary exploration of preparing ultrafine ZPT particles via a liquid–liquid microchannel reactor, we separately investigated the individual effects of feed flow rate, initial reactant concentration and microchannel size on particle size and micromorphology. This approach efficiently narrows down feasible parameter ranges, screens out dominant factors and reduces experimental workload at the early stage, laying a foundation for subsequent statistical experimental design to analyze factor-to-factor interactions. Meanwhile, we acknowledge inherent limitations of the OFAT method. Only one variable is adjusted while other parameters remain constant throughout OFAT experiments, which ignores interactive effects among different operating variables. For the microchannel-based reaction system, feed flow rate, initial reactant concentration and microchannel size mutually restrict one another. Consequently, the optimal conditions derived from OFAT are likely a local optimum rather than a global optimum across the full parameter space. Statistical design of experiments will be adopted in our follow-up work to explore variable interactions and obtain globally optimized parameters.

## 2. Results and Discussion

Uniformly dispersed ultrafine ZPT particles with reduced particle size were successfully fabricated via rapid precipitation reaction in microchannel reactors. The experimental conditions were systematically optimized, experimental phenomena and data were comprehensively analyzed, and suitable drying methods were further screened and evaluated.

### 2.1. XRD Characterization of Synthesized ZPT Products

**Figure 1:**
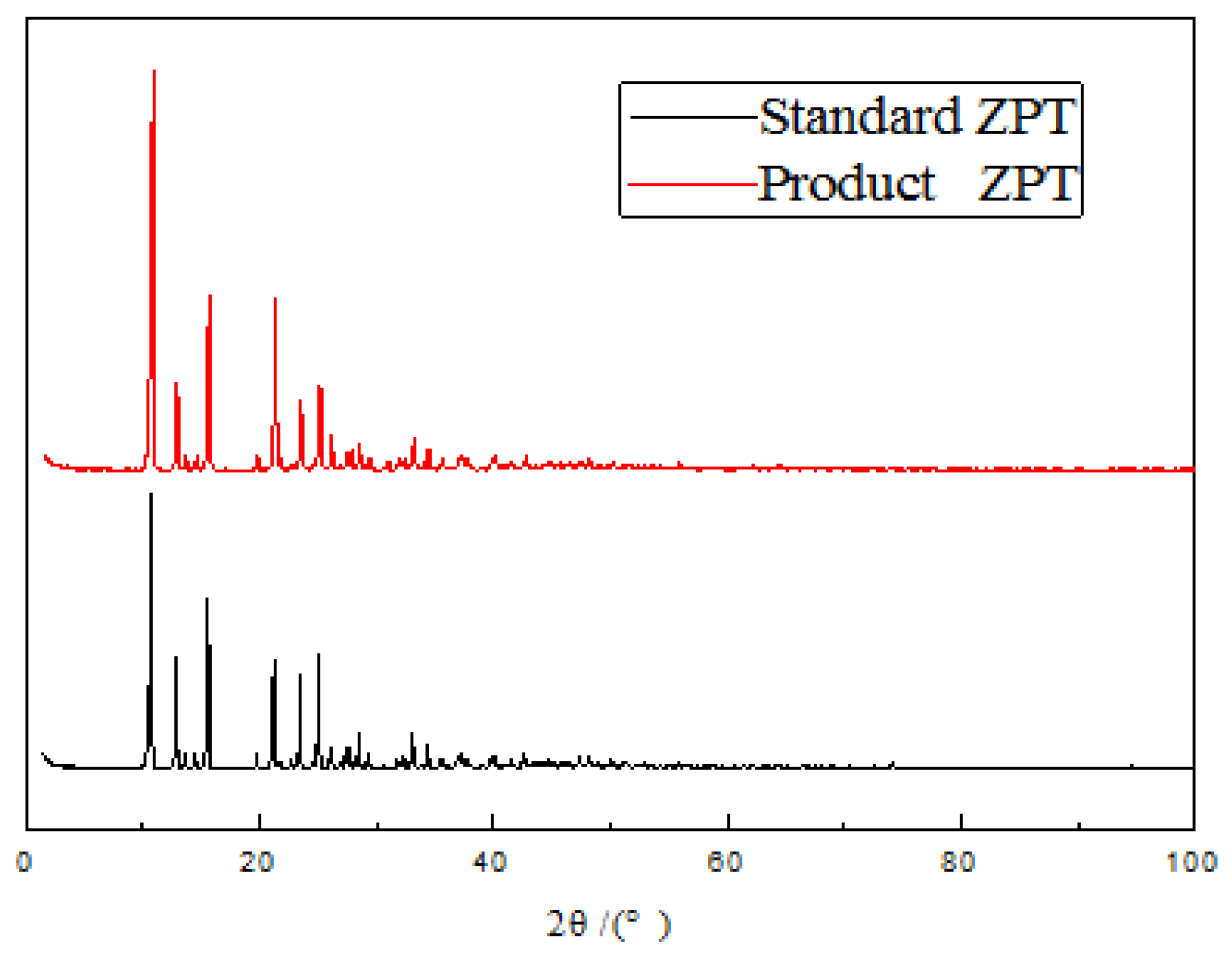
XRD comparison patterns between as-prepared ZPT and standard ZPT.

ZPT ultrafine particles were prepared via SPT-based rapid precipitation in microchannel reactors. Typical samples were selected for XRD detection, and the obtained diffraction patterns were compared with standard ZPT reference spectra, as displayed in Figure 1.

The peaks at 2θ ≈ 7.5°, 12.5°, 19.0° and 22.0° are the main characteristic peaks of ZPT, and the peak at 7.5° is the strongest one. By comparing the XRD patterns of experimentally synthesized ZPT with standard ZPT spectra, an excellent consistency can be clearly observed for all diffraction peaks. The result confirms that the target product is successfully synthesized zinc pyrithione with high chemical purity.

### 2.2. Effect of Material Flow Velocity in Microchannel

The material flow velocity reflects the flow behavior of reactants inside microchannels. A higher flow velocity enables the fluid flow to approach plug flow and effectively weakens the backmixing of reactants. Meanwhile, increasing flow velocity enhances the collision intensity between two reactant streams and optimizes the micromixing efficiency. These advantages facilitate the acquisition of precipitated particles with smaller particle size and narrower size distribution.

**Figure 2:**
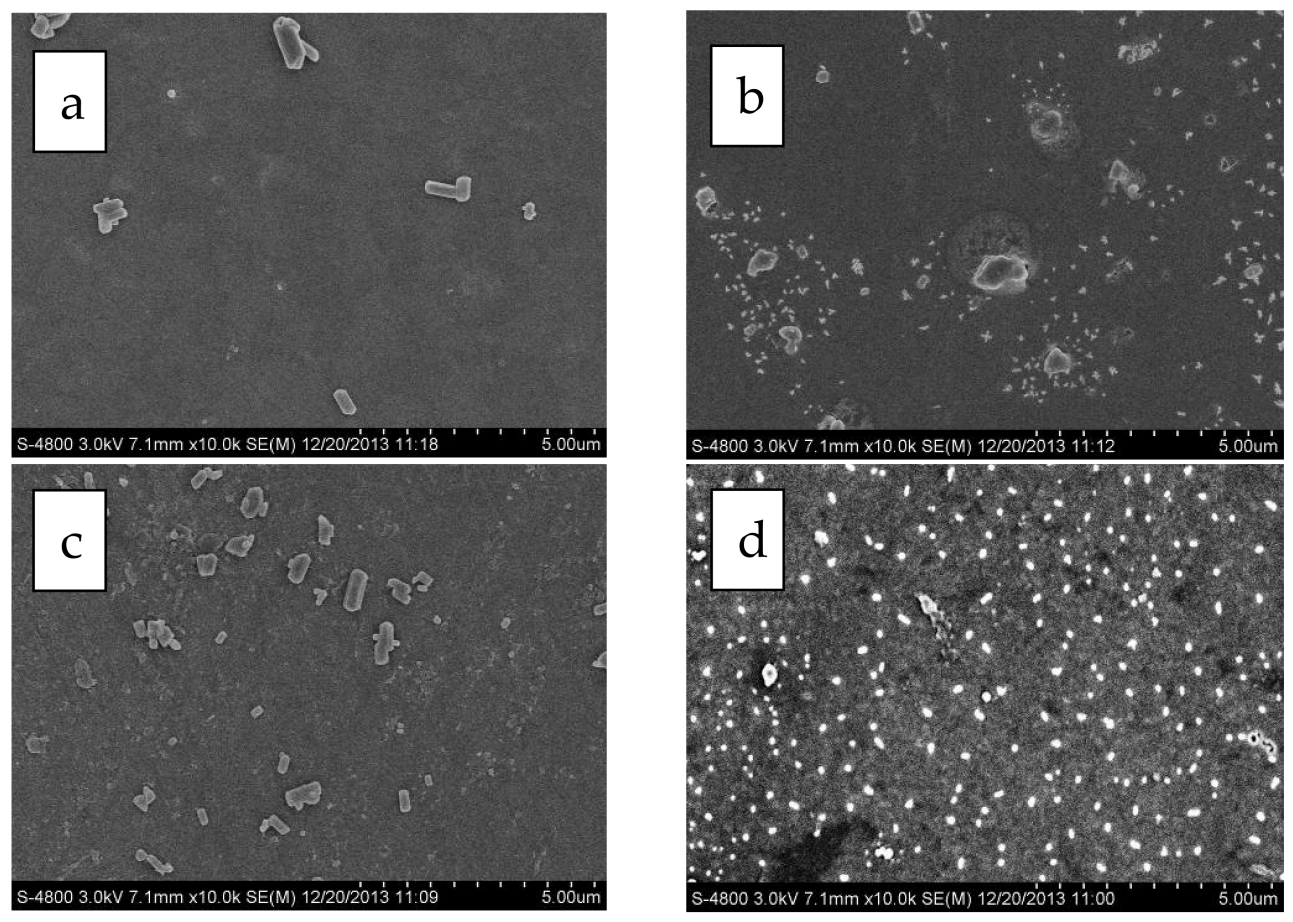
SEM images of ultrafine ZPT particles prepared at different flow velocities. (**a**) microchannel flow velocities of 8.7 m/s; (**b**) microchannel flow velocities of 13 m/s; (**c**) microchannel flow velocities of 17.4 m/s; (**d**) microchannel flow velocities of 21.7 m/s.

In this experiment, the concentrations of sodium pyrithione (SPT) and zinc sulfate were fixed at 0.5 mol/L and 0.25 mol/L respectively, with the reaction performed at room temperature under 1 MPa pressure. The flow rates were adjusted to 10 L/h, 15 L/h, 20 L/h and 25 L/h by rotameter, corresponding to microchannel flow velocities of 8.7 m/s, 13 m/s, 17.4 m/s and 21.7 m/s. Ultrafine ZPT particles were prepared under the above four velocity conditions, and the corresponding SEM images are presented in Figure 2a–d.

As shown in Figure 2, at the same magnification, the particle size of ZPT decreases gradually and the particle size distribution becomes more uniform with the increase in flow velocity. When the maximum flow velocity reaches 21.7 m/s, the particle size is approximately 100 nm with uniform morphology and consistent particle dimension.

During the reaction process in microchannels, two kinds of reactant solutions flow at specific rates driven by external power and collide with each other to synthesize ZPT particles. High flow velocity generates strong shear force via high-speed directional collision, which realizes highly uniform mixing of SPT molecules and ions in zinc sulfate solution, thus endowing the obtained ZPT particles with excellent uniformity. Additionally, the residence time of ZPT emulsion in microchannels is only at millisecond level. All reactants can reach ideal supersaturation state rapidly, and the newly formed products are promptly delivered into storage tanks. Consequently, ultrafine ZPT particles with small size and narrow size distribution can be successfully fabricated under high flow velocity conditions.

Compared with the ZPT particles prepared at the maximum flow velocity in the research reported by Zhao Hua [10], the samples obtained in this work possess superior comprehensive performance, including smaller particle size, better morphological uniformity and narrower particle size distribution. This superiority is mainly attributed to the higher reaction flow velocity adopted in the present study, which verifies that high flow velocity is conducive to the synthesis of high-quality particles.

In terms of research methodology, the flow velocity regulation strategy in this work is distinctly different from the injection precipitation method for pyrithione salts proposed by Luo Peicheng [11]. The relevant patent only described that the feed liquid enters the reactor through an injector without specifying specific injection velocity parameters, leading to invisible flow field conditions and uncontrollable particle size regulation during preparation. Furthermore, the average particle size of ZPT prepared in this study is around 150 nm, which is much smaller than the 300 nm particles obtained by Zhao Fengyun et al. [6]. The core reason lies in that traditional tank reactors rely on mechanical stirring to achieve solution mixing, whose mixing efficiency is far lower than the high-speed collision mixing effect inside microchannels. Accordingly, the ZPT particles prepared via the optimized microchannel strategy exhibit superior physicochemical properties including smaller particle size and more uniform morphology, which endows the products with favorable basic conditions for subsequent functional application research.

### 2.3. Effect of Initial Concentration of Reactants

The initial concentration of reactant solutions not only affects the reaction rate, but also determines the quality of final products. At relatively low solution concentrations, ions fail to reach effective supersaturation, and unreacted ions tend to adsorb on the surface of primary particles, which further promotes particle growth. In contrast, excessively high concentrations lead to excessive supersaturation of the solution and increase the collision probability among crystal nuclei, also resulting in enlarged particle size. Therefore, the initial reactant concentration plays a critical role in the preparation of ZPT particles via rapid precipitation method.

**Figure 3:**
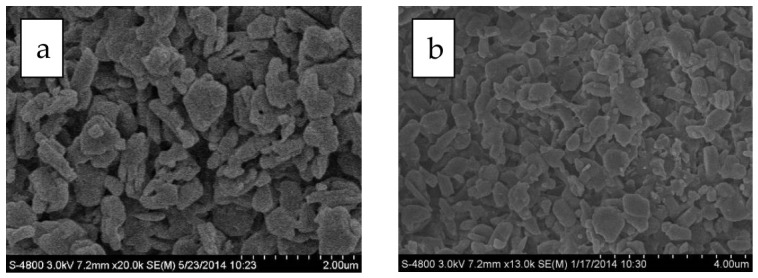

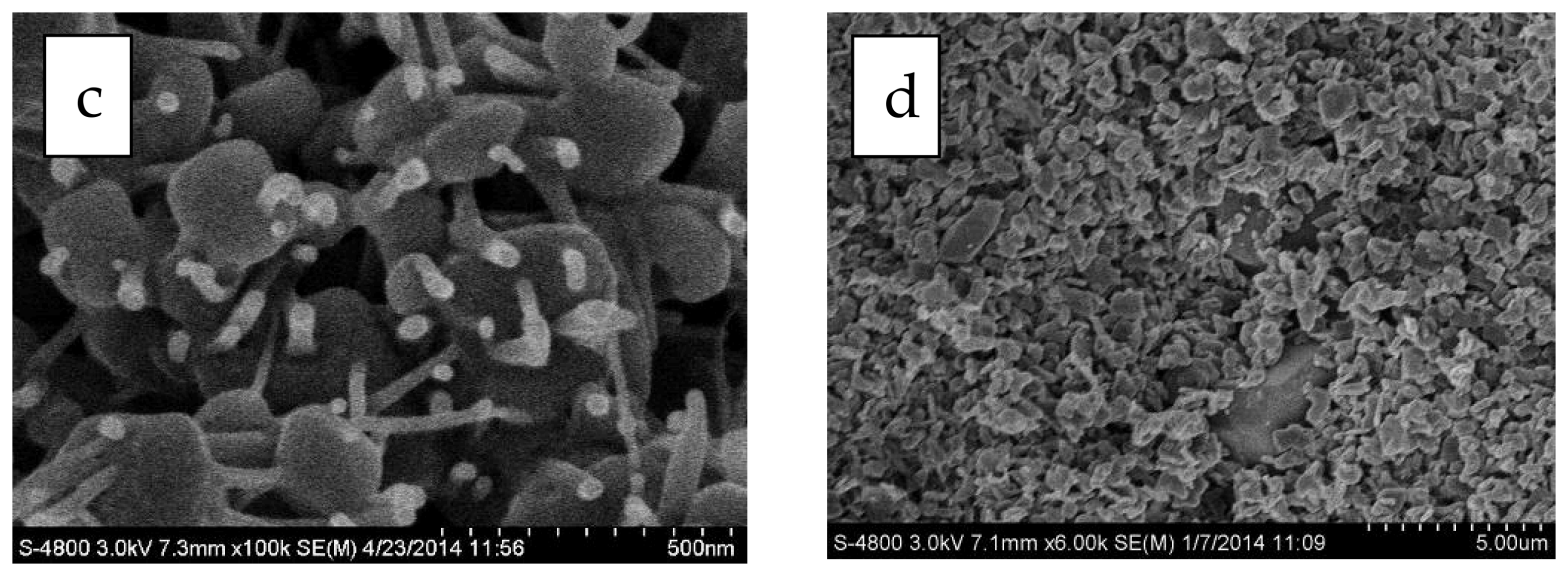
SEM images of ultrafine ZPT particles prepared at different reactant solution concentrations. (**a**) SPT 0.25 mol/L; (**b**) SPT 0.40 mol/L; (**c**) SPT 0.50 mol/L; (**d**) SPT 1.00 mol/L.

**Figure 4:**
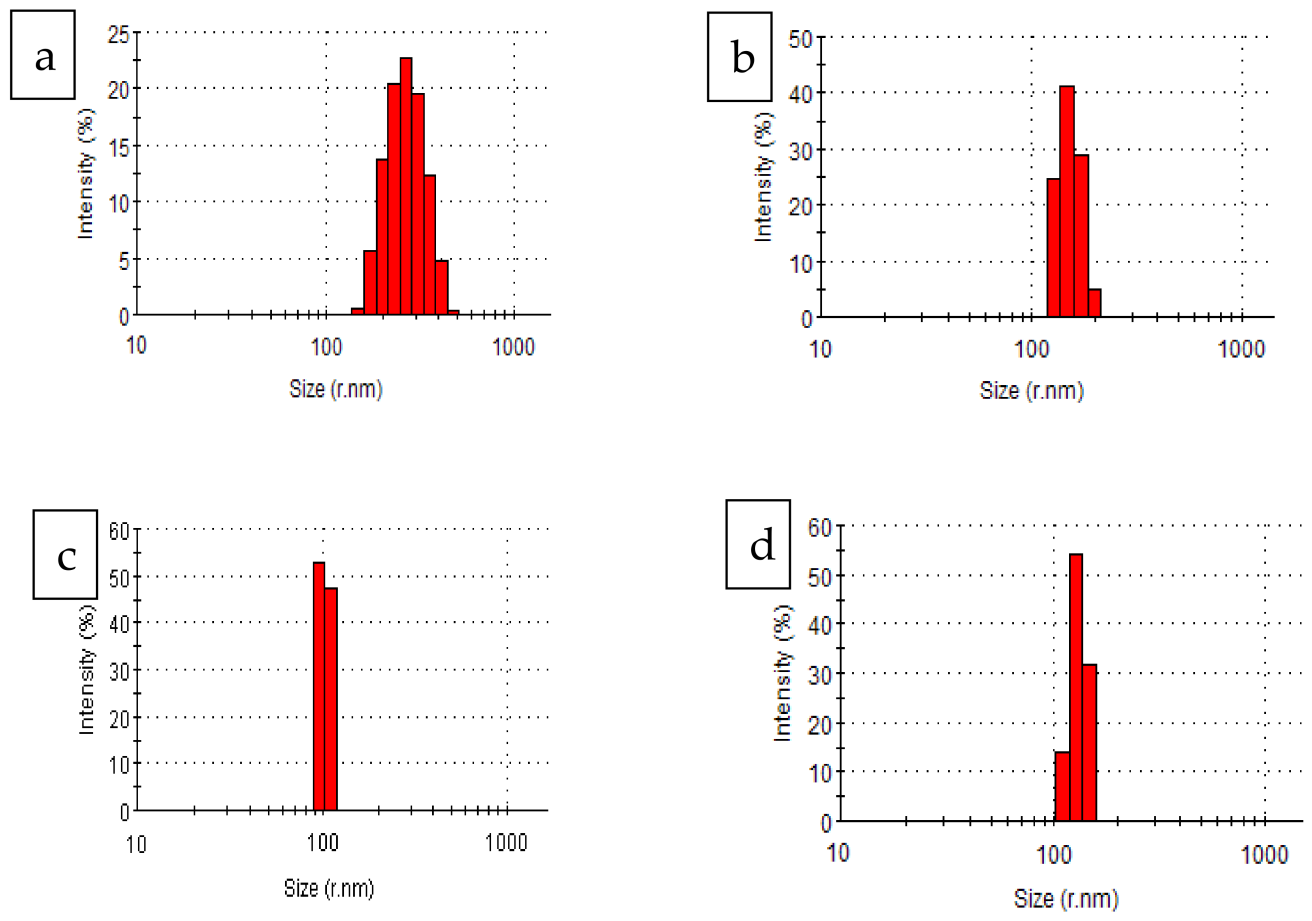
Particle size distribution curves of ultrafine ZPT particles prepared at different reactant concentrations. (**a**) SPT 0.25 mol/L; (**b**) SPT 0.40 mol/L; (**c**) SPT 0.50 mol/L; (**d**) SPT 1.00 mol/L.

Four groups of reactant solutions with different concentrations were prepared as listed in Table 1. All experiments were performed at room temperature under the pressure of 1 MPa, and the flow rate was fixed at 25 L/h, corresponding to a solution flow velocity of 21.7 m/s. Ultrafine ZPT particles were synthesized under four different concentration conditions. The particle size and uniformity data are summarized in Table 2, the corresponding SEM images are shown in Figure 3a–d, and the particle size distribution curves measured by Malvern laser particle size analyzer are presented in Figure 4.

It can be observed from Figure 3 and Figure 4 and Table 2 that Group c yields ultrafine ZPT particles with smaller particle size and narrower size distribution. In this experiment, identical liquid flow velocity was maintained, so equivalent shear force was generated among solutions with different initial concentrations during the reaction process.

At low reactant concentrations (Group a and Group b), the ionic supersaturation degree is insufficient, leading to a small number of crystal nuclei. Unreacted ions tend to adsorb on the surface of newly formed ZPT particles, which eventually increases the final particle size. By contrast, excessively high concentration (Group d) accelerates the nucleation rate and raises the particle number density per unit volume. Numerous fine crystal embryos with high chemical potential are thermodynamically unstable, resulting in partial dissolution and further growth of crystals, which accounts for the formation of large-sized grains at high concentrations.

Comparative analysis with the results reported by Zhao Fengyun reveals that ZPT particles synthesized in microchannel reactors exhibit better uniformity and smaller particle size than those prepared in traditional tank reactors under the same concentration conditions. This superiority is attributed to the miniature channel dimension and short material residence time of microchannel reactors, which greatly weaken the influence of ionic supersaturation compared with conventional tank reaction systems.

### 2.4. Effect of Microchannel Dimension

Microchannel reactors possess distinctive mass and heat transfer performances, which can markedly elevate the production capacity per unit time and unit volume. Under identical flow velocity and reaction duration, reactors with larger channel sizes can achieve higher product output, which is favorable for satisfying the production demand of zinc pyrithione in industrial scale-up. Nevertheless, the expansion of channel dimension may alter the flow pattern and micromixing efficiency inside the channels, thereby exerting crucial influences on the quality of final products. Accordingly, the correlation between microchannel size and particle morphology was systematically investigated in this work.

**Figure 5:**
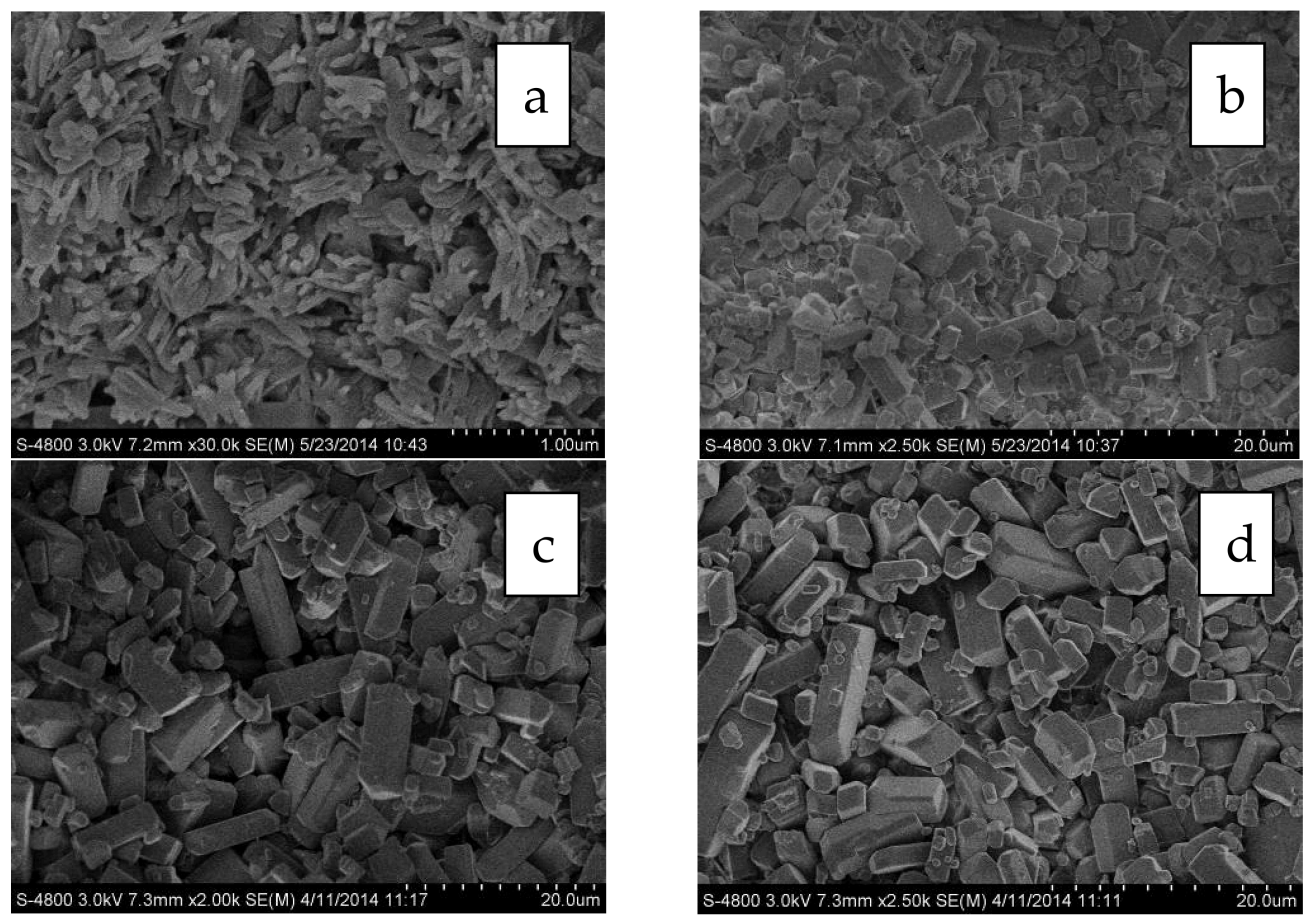
SEM images of ZPT ultrafine particles prepared with different reaction channel sizes. (**a**) 0.8 mm; (**b**) 2 mmL; (**c**) 3 mm; **(d**) 6 mm.

In this section, the concentrations of sodium pyrithione (SPT) and zinc sulfate were fixed at 0.5 mol⋅L^−1^ and 0.25 mol⋅L^−1^ respectively. All experiments were conducted at room temperature with a constant pressure of 1 MPa. Four groups of microchannels with different inner diameters (listed in Table 3) were adopted, and the flow velocity of reaction solutions was uniformly controlled at 21.7 m/s throughout the tests. Ultrafine ZPT particles were successfully prepared under different channel sizes. The corresponding SEM images are displayed in Figure 5a–d, and the detailed experimental data are summarized in Table 4.

Microchannel reactors possess outstanding mass-transfer and heat-transfer performance, which greatly improves productivity per unit volume. From the industrial-scale perspective, increasing channel hydraulic diameter can enhance overall throughput under identical linear velocity, which is beneficial for large-scale ZPT production. However, enlarging channel dimensions changes flow patterns and micromixing efficiency, alters supersaturation distribution and further regulates nucleation and crystal-growth processes. Dimensionless parameters including Reynolds number (Re) and Damköhler number (Da) are introduced to reveal the underlying mechanism quantitatively.

In this work, the concentrations of SPT solution and zinc-sulfate solution are fixed at 0.5 mol/L and 0.25 mol/L, respectively. The experiments are performed at room temperature and 1 MPa. The linear flow velocity is maintained at 21.7 m/s, and four groups of microchannels with different hydraulic diameters are adopted (Table 3). The two key dimensionless numbers are calculated as follows:

$$Re=\frac{\rho ud_{h}}{\mu };Da=\frac{\tau_{mix}}{\tau_{reaction}}$$

where ρ = fluid density, u = linear velocity, $d_{h}$ = hydraulic diameter of microchannel, μ = dynamic viscosity, $\tau_{mix}$ = mixing time, and $\tau_{reaction}$ = reaction time. The Reynolds number evaluates the ratio of inertial force to viscous force, while the Damköhler number compares mixing time against reaction time. When Da>1, crystallization is mixing-controlled.

According to SEM images (Figure 5) and particle-size data in Table 4, the sample from the 0.8 mm channel shows smaller particle size and narrower particle-size distribution owing to higher Re. Short diffusion distance inside the narrow channel shortens $\tau_{mix}$ and increases Da. A uniform high-supersaturation field is formed to trigger intensive nucleation. Even though the linear velocity is fixed at 21.7 m/s, larger hydraulic diameter reduces Re and Da. Longer diffusion distance increases local mixing time, and $\tau_{mix}$ exceeds $\tau_{reaction}$ in some regions. Poor micromixing leads to uneven supersaturation and low nucleation rate. Unreacted ions continuously deposit onto formed nuclei, which finally generates larger ZPT particles with worse uniformity.

In brief, a larger-sized channel improves throughput but reduces Re and Da and weakens micromixing. Excessively narrow channels bring about channel blockage and low productivity. Therefore, the number-up strategy by parallel arrangement of small-size microchannels is recommended for industrial-scale production to balance mixing quality and production capacity.

### 2.5. Performance Analysis of ZPT Products

#### 2.5.1. Zeta Potential Analysis of ZPT Particles

Zeta potential is a measure of the strength of repulsive or attractive forces between particles. Its core significance lies in the correlation between its value and the stability of colloidal dispersion. The smaller the molecules or dispersed particles, the higher the absolute value of Zeta potential, and the more stable the system is. Such particles can resist aggregation during dissolution and dispersion and present excellent dispersibility. On the contrary, a lower absolute value of Zeta potential makes particles prone to coagulation and agglomeration. In this case, intermolecular attractive forces outweigh repulsive forces, destroying the dispersion state and resulting in particle agglomeration.

The colloidal stability can be determined by comparing the measured Zeta potential values of particles with the data in Table 5. Zeta potentials of ZPT particles prepared under different flow rates, different initial reactant concentrations and different reaction channel sizes were tested respectively. The test results are shown in Table 6, Table 7 and Table 8.

It can be seen from the data in Table 6, Table 7 and Table 8 that particles synthesized at low flow rates exhibit poor stability. As the flow rate rises, the anti-agglomeration capacity improves and the stability reaches a moderate level. Particles prepared with the initial SPT concentration of 0.5 mol/L possess favorable stability. Channel size exerts slight influence on zeta potential. Secondary aggregated particles formed in large channels show better stability than primary particles. The stability evaluation based on zeta potential results is consistent with SEM observation findings. In this work, the reaction pH was consistently maintained at 6.5. Under this experimental condition, deprotonated pyrithionate anions (PT^−^) originating from SPT are dominantly adsorbed on ZPT crystal surfaces and provide negative surface charge, whereas OH^−^ ions only exert a minor effect. Synthesis-related parameters influence zeta potential mainly by adjusting the adsorption amount of PT^−^ ions. Higher flow velocity enhances micromixing, which makes PT^−^-ions distribute uniformly and facilitates sufficient ion adsorption, leading to higher absolute zeta-potential values. Increased channel size weakens local mixing and causes uneven consumption of PT^−^ anions, which reduces PT^−^ adsorption on partial crystal surfaces and decreases absolute zeta-potential values. Varied reactant-concentration changes residual PT^−^ content in bulk solution and further affects surface-adsorption behaviors.

#### 2.5.2. TGA of ZPT Particles

Thermogravimetric analysis (TGA) measures the mass variation in samples with temperature under programmed heating conditions. It is applied to investigate thermal behaviors and further characterize material properties.

**Figure 6:**
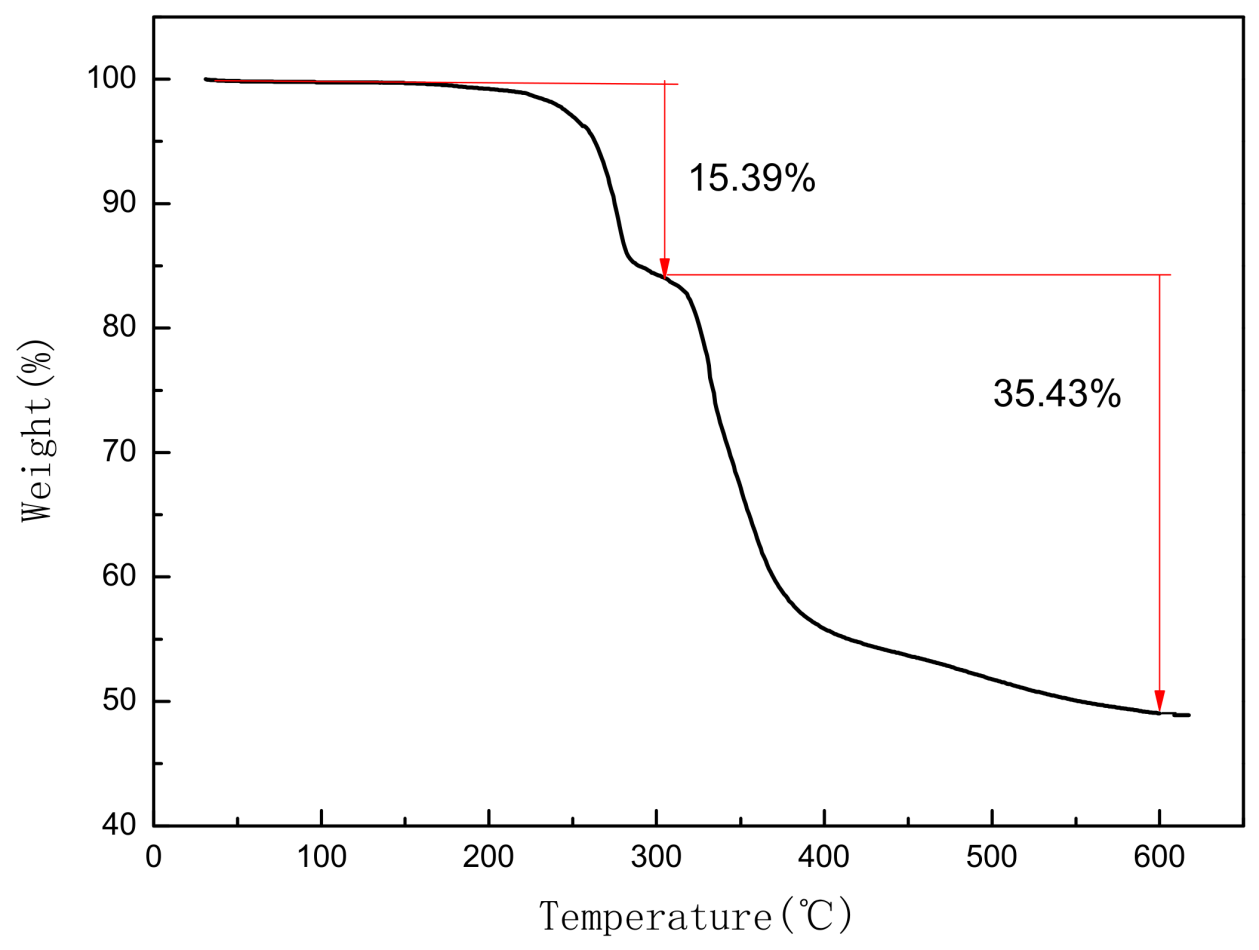
Thermogravimetric analysis curve of ZPT product.

Though ZPT serves as a bacteriostatic and mildewproof agent and works at ambient temperatures with low requirement on thermal stability, it often undergoes thermal processing after being incorporated into other solution systems. Hence favorable thermal stability within processing temperature range is essential. A powdered ZPT sample obtained from one set of dried products was tested via thermogravimetric analyzer. The results are presented in Figure 6.

It can be observed from the TGA curve that mass loss initiates at 240 °C. A prominent primary mass loss of 15.39% occurs at 280 °C, which is attributed to the release of sulfur dioxide during decomposition. As temperature rises to 600 °C, the total mass loss reaches 35.43%, mainly resulting from carbon dioxide emission. Accordingly, the ambient temperature should be kept below 200 °C to maintain the stability of ZPT in practical application.

### 2.6. Drying Methods of ZPT

**Figure 7:**
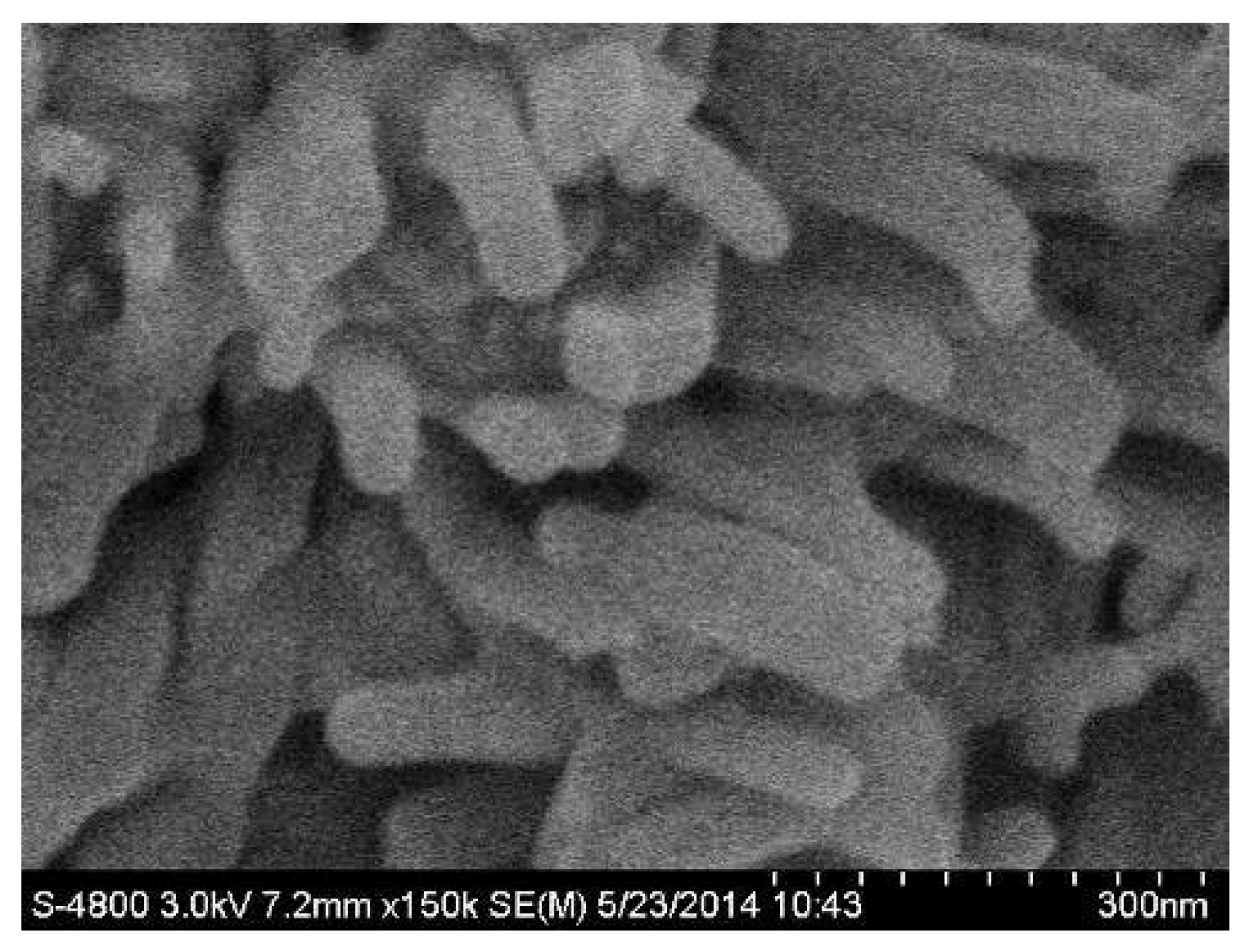
SEM image of ZPT particles before drying treatment.

Dry ZPT powder is required in many application scenarios of ultrafine ZPT particles, for instance serving as mildew inhibitor in paint. Wet ZPT needs dehydration treatment to obtain powdery particles. Nevertheless, ultrafine particles possess size effect, surface effect, surface electronic effect and short-range effect, which easily lead to particle agglomeration during drying. The aggregated particles cannot satisfy practical application demands. Therefore, drying methods of ZPT were screened and optimized in this paper. Figure 7 presents the SEM image of original ZPT particles, taken as the reference for evaluating subsequent drying effects.

#### 2.6.1. Static Heating Drying

**Figure 8:**
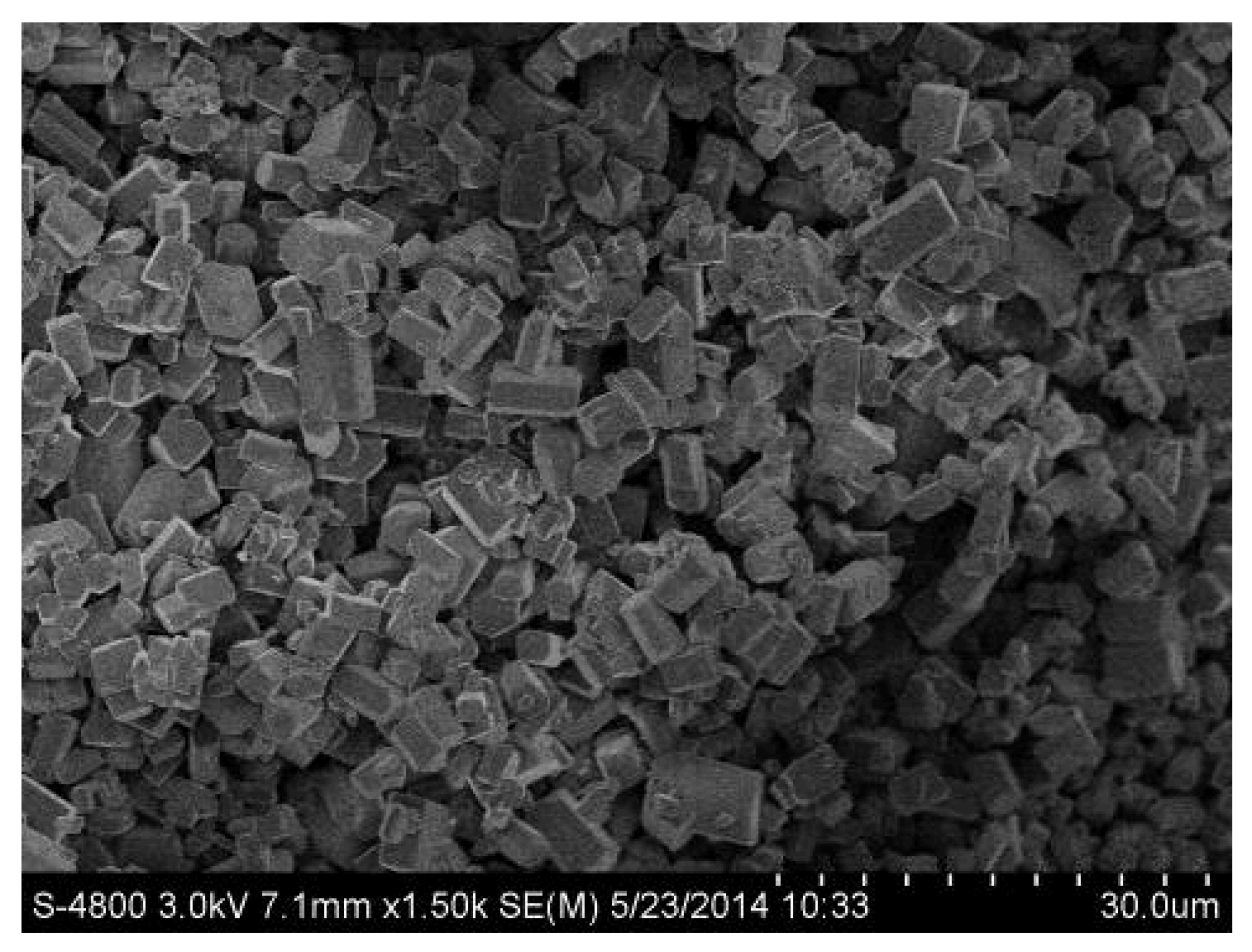
SEM image of ZPT products obtained by static heating drying method.

Static heating drying is performed with an electric blast drying oven. Electric heating and circulating hot air ensure even temperature distribution inside the chamber, which is a typical static drying method. It is widely used in chemical, pharmaceutical, foundry, automobile, food and mechanical industries. The prepared ZPT particles were dehydrated in the oven, and the dried powder was characterized by SEM. The results are shown in Figure 8.

Comparative images show that the particle size increases from 180 nm before drying to 6 μm after oven drying, transforming from nanometer scale to micrometer scale. During drying, samples remain static. Moisture evaporates only under high temperature. After surface dehydration, residual internal water migrates outward, strengthening hydroxyl bonding between particles and triggering agglomeration. In addition, particle shape changes from strip to three-dimensional prism. Hence, static heating drying is not applicable for ZPT particles.

#### 2.6.2. Static Freeze Drying

**Figure 9:**
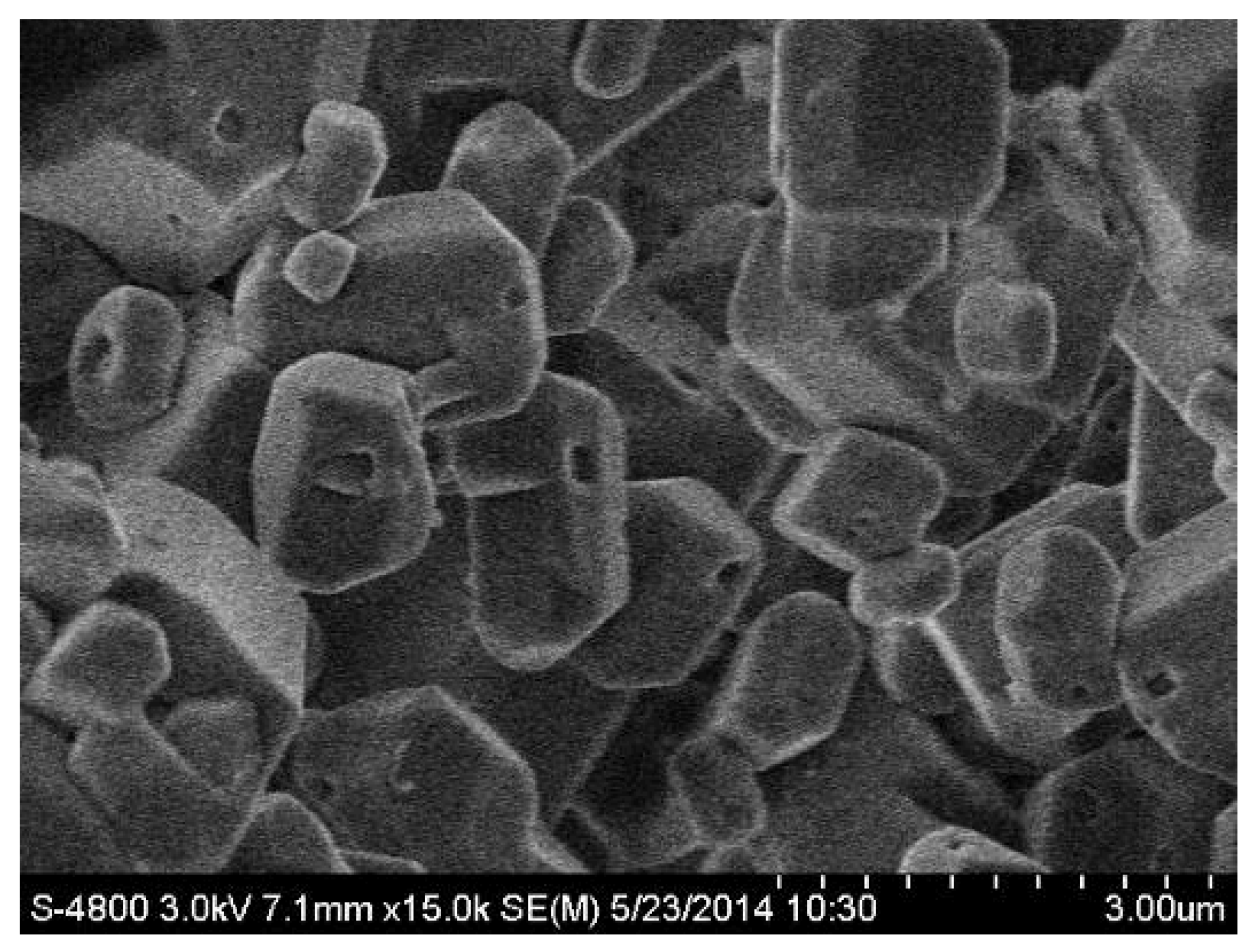
SEM image of ZPT products obtained by freeze drying method.

Freeze dryer is adopted for this method, which consists of refrigeration, vacuum, heating and electric control systems. Based on sublimation principle, samples are rapidly frozen at low temperature. Frozen moisture directly sublimates into vapor and escapes under vacuum condition. This method also belongs to static drying. The frozen ZPT particles were dehydrated in freeze drying chamber. The SEM image of dried samples is shown in Figure 9.

It can be seen from Figure 9 that the particle size after freeze drying is 1.5 μm with uniform particle size distribution. Compared with commercial products, the obtained ZPT powder has a smaller and more uniform particle size. As another static drying technique, freeze drying achieves a far better effect than oven drying. Water is removed via sublimation, which weakens the action of surface hydroxyl groups and intermolecular hydrogen bonds. The dried ZPT presents a spongy and porous structure with nearly unchanged volume, proving no concentration phenomenon occurs during dehydration. Nevertheless, the particle size still fails to meet the required nanoscale standard. Limited processing capacity and discontinuous operation also restrict its application in large-scale industrial production.

#### 2.6.3. Dynamic Spray Drying

**Figure 10:**
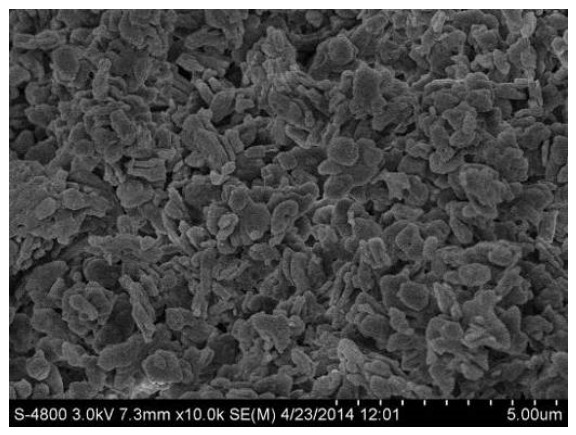
SEM image of ZPT products obtained by spray drying.

A spray dryer is used for this drying process. It serves to dry, separate and recover materials. Raw materials are atomized into tiny droplets with large specific surface area. Rapid water evaporation occurs upon contact with hot air, yielding dry products with excellent dispersibility and fluidity in a short time. This dynamic drying technique supports continuous large-scale production. The SEM image of ZPT particles obtained by spray drying is displayed in Figure 10.

It can be observed that ZPT particles prepared by spray drying are sheet-shaped with uniform particle size of 600 nm. Comparison of three drying methods indicates spray drying is suitable for ZPT ultrafine particles. The obtained dry powder exhibits good performance to commercial products.

SEM analysis of the dried products reveals that the obtained particles exhibit relatively regular sheet-like morphology with a small average particle size. It demonstrates that the microchannel-reactor-based method can effectively restrain excessive grain growth by optimizing hydrodynamic conditions. Although slight surface agglomeration arises during drying, the intrinsic sheet-shaped outline of primary particles is well-preserved. By contrast, samples synthesized via conventional stirring suffer from severe agglomeration and over-growth of primary particles owing to the absence of efficient nucleation control. Consequently, the original grain size and geometric morphology can hardly be identified. SEM images of dried samples further illustrate their evolutionary behaviors upon high-temperature treatment. Both samples undergo particle growth and partial melting to a certain extent. Products from the microchannel reactor maintain smaller overall particle sizes with narrow size distribution, and most particles retain spherical features. In comparison, particles prepared by conventional fully stirred reactors display severe agglomeration, irregular morphology, wide particle-size variation and poor structural uniformity.

## 3. Materials and Methods

### 3.1. Experimental Reagents

The main reagents adopted in this experiment are listed in Table 9.

### 3.2. Experimental Equipments

Main instruments applied for ZPT preparation via the SPT method are summarized in Table 10.

### 3.3. Preparation of ZPT

ZPT was synthesized via the SPT method [12] in this study. Reaction solutions with designated concentrations were prepared firstly, then emulsion containing ZPT was obtained by microchannel reactor. After water washing and centrifugal sedimentation, the filter cake was diluted with water to prepare ZPT suspension, or dehydrated in a dryer to obtain ZPT powder. The chemical reaction involved is presented as follows:


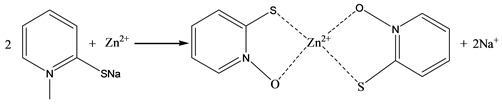


#### 3.3.1. Experimental Process

**Figure 11:**
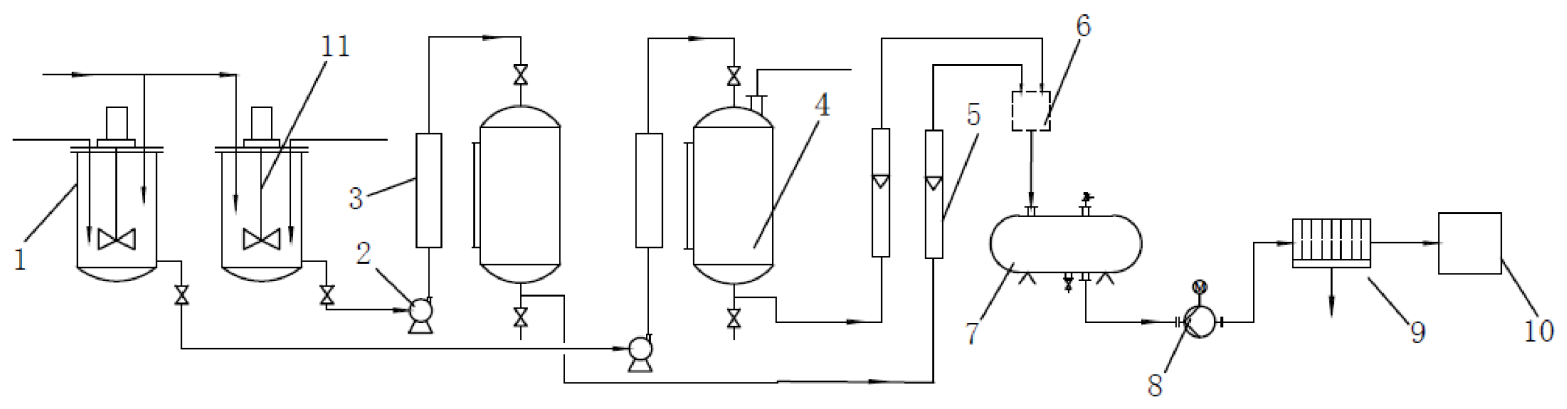
Flow chart of ZPT preparation. 1. SPT Raw material mixing tank; 2. Feed pump; 3. Solution purifier; 4. Reaction liquid storage tank; 5. Rotameter; 6. Microchannel reactor (φ 0.8–6 mm); 7. Product storage tank; 8. Product delivery pump; 9. Filter; 10. Dryer; 11. ZnSO_4_ Raw material mixing tank.

Figure 11 shows the experimental flow chart of ZPT preparation.

#### 3.3.2. Experimental Procedures

The SPT (0.25–1 mol/L) and ZnSO_4_ (0.125–0.5 mol/L) reaction solutions of a certain concentration were stored in respective reaction liquid storage tanks. The partial pressure valve of nitrogen cylinder was adjusted to maintain stable pressure. The opening of rotameter was tuned to set required flow rate. The reaction solutions flowed into the microchannel reactor at fixed velocity and rapidly reacted to form emulsion containing ZPT particles. The emulsion was collected in a product tank, then subjected to repeated centrifugal filtration and water washing. The obtained filter cake was dried to acquire ZPT powder.

1. Preparation and purification of raw liquid: Calculate raw material dosage for target concentration; weigh and dissolve in distilled water with stirring. The mixed solution was filtered to remove impurities. Store the prepared reaction solution separately in the reaction solution storage tanks.
2. ZPT synthesis in microchannel reactor: By adjusting the rotor flowmeter and controlling the flow rate of the reaction material, the material reacts in the microchannel reactor to obtain emulsions containing ZPT under different conditions. Prepare reactants of different concentrations, control different reactant flow rates, and change the size of microchannel reactors to obtain different product data.
3. Preparation of dry ZPT powder: The emulsion was treated by centrifugal filtration and water washing. The filter cake was dried with an electric blast dryer, fluidized bed dryer, freeze dryer and spray dryer respectively to obtain final ZPT product powder.

### 3.4. Characterization Methods

#### 3.4.1. X-Ray Diffraction Analysis

ZPT ultrafine particles were characterized by Rigaku D/max 2500 v/pc X-ray powder diffractometer. Test conditions: CuKα radiation, graphite monochromator, tube current 150 mA, tube voltage 40 kV, scanning range 2θ = 1.5°~100°. The Scherrer formula D = Kλ/Bcosθ was applied to calculate particle size based on diffraction peak half-width B and diffraction angle θ.

#### 3.4.2. Scanning Electron Microscopy Analysis

A Hitachi S4800-I scanning electron microscope was adopted for sample characterization. Operating voltage: 3 kV; minimum resolution: 1 nm at 15 kV, 1.4 nm at 1 kV; elemental analysis scope: Be4-U92; magnification: 20~800,000 times.

#### 3.4.3. Particle Size Distribution Analysis

A Nano-S90 laser particle size analyzer (Malvern Instruments Ltd., Malvern, UK) was used to test particle distribution. Technical parameters: measuring range 2 nm~3 μm, sample temperature control 2~90 °C, minimum injection volume 20 μL.

#### 3.4.4. Zeta Potential Analysis

A Nano-Z zeta potential analyzer (Malvern Instruments Ltd., Malvern, UK) was employed for measurement. Parameters: particle size range 0.3 nm~10 μm, concentration range 0.1 ppm~40% *w*/*v*, detection angles 175° and 12.8°, minimum sample volume 12 μL.

#### 3.4.5. Thermogravimetric Analysis

A Q600 simultaneous thermal analyzer (TA Instruments, New Castle, DE, USA) was used for the thermal performance test. The sample was heated from room temperature to 600 °C at a heating rate of 10 °C/min under nitrogen atmosphere.

## 4. Conclusions

ZPT ultrafine particles were synthesized via the SPT method in a microchannel reactor. Experimental parameters were compared and optimized. The optimal conditions for smaller particle size and uniform distribution were determined: SPT concentration 0.5 mol/L, zinc sulfate concentration 0.25 mol/L, flow velocity 21.7 m/s, channel size 0.8 mm, room temperature and pressure 1 MPa. Under such conditions, the particle size of wet ZPT reached 180 nm, decreasing by 40% compared with tank reactor, with a PDI value of 0.18. Static and dynamic drying methods were screened and optimized. Spray drying effectively alleviates particle agglomeration and supports continuous production, which is a feasible drying technique for wet ZPT particles.

## Figures and Tables

**Table 1:** List of four different initial concentrations of reactant solutions.

No.	Concentration of SPT (mol/L)	Concentration of ZnSO_4_ (mol/L)
a	0.25	0.13
b	0.40	0.20
c	0.50	0.25
d	1.00	0.50

**Table 2:** List of particle products prepared with 4 different initial reactant solutions.

No.	Particle Size (nm)	Uniformity
a	600	Non-uniform
b	900	Relatively uniform
c	190	Relatively uniform
d	1000	Relatively uniform

**Table 3:** List of four different channel inner diameters.

No.	Inner Diameter of Reaction Channel (mm)
a	0.8
b	2
c	3
d	6

**Table 4:** Morphological characteristics of particles prepared with four different channel sizes.

No.	Particle Size (nm)	Uniformity	Particle Morphology
a	220	Relatively uniform	Fingered morphology
b	2000~6000	Non-uniform	Prismatic morphology
c	2000~8000	Non-uniform	Prismatic morphology
d	2000~12,000	Non-uniform	Prismatic morphology

**Table 5:** Relationship between different potential values and colloidal stability.

Zeta Potential (mV)	Colloidal Stability
Absolute value 0~±10	Rapid coagulation
Absolute value ±10~±30	Gradually unstable
Absolute value ±30~±40	General stability/Good stability/Excellent stability
Absolute value ±40~±60	General stability/Good stability/Excellent stability
Absolute value above ±61	General stability/Good stability/Excellent stability

**Table 6:** Potential values of ZPT particles under different material flow rates in microchannel.

Flow Rates (L/h)	Zeta Potential (mV)
10	−10.4
15	−13.8
20	−15.7
25	−20.6

**Table 7:** Potential values of ZPT particles prepared at different initial reactant concentrations.

Reactant Concentrations (Calculated as SPT) (mol/L)	Zeta Potential (mV)
0.25	−14.1
0.4	15.5
0.5	−20.2
1	−12.4

**Table 8:** Potential values of ZPT particles prepared under different channel sizes.

Microchannel Dimension (mm)	Zeta Potential (mV)
0.8	−19.9
2	−19.1
3	−21.8
6	−20.7

**Table 9:** List of experimental raw materials.

Reagent Name	Specification	Manufacturer
SPT	40.65%	Wuxi Zhufeng Fine Chemical Co., Ltd., Wuxi, China
ZnSO_4_·7H_2_O	Industrial Grade	Xinji Jiatong Chemical Co., Ltd., Xinji, China
Distilled Water	Double-distilled	Self-prepared
N_2_	99.999%	Xisanjiao Oxygen Station, Shijiazhuang, China

**Table 10:** List of experimental equipment.

Equipment Name	Model	Manufacturer
Electronic Analytical Balance	JJ200B	Changshu Shuangjie Testing Instrument Factory, Changshu, China
Raw Material Preparation Tank	—	Self-made
Rotameter	LZB-4F	Yuyao Industrial Automation Instrument Factory, Yuyao, China
Stainless Steel Microchannel Reactor	—	Self-made
Electric Blast Drying Oven	101-2AB	Tianjin Taisite Instrument Co., Ltd., Tianjin, China
Freeze Dryer	LGJ-10	Beijing Songyuan Huaxing Technology Development Co., Ltd., Beijing, China
Spray Dryer	—	Beijing Research Institute of Chemical Industry, Beijing, China
Fluidized Bed Dryer	—	Self-made
X-ray Diffractometer	2500 v/pc	Rigaku Corporation, Akishima, Japan
Field Emission Scanning Electron Microscope	S4800-I	Hitachi Limited, Tokyo, Japan
Laser Particle Size Analyzer	Nano-S90	Malvern Panalytical, Malvern, UK
Zeta Potential Analyzer	Nano-S90	Malvern Panalytical
Simultaneous Thermal Analyzer	Q600	TA Instruments, New Castle, DE, USA

## Data Availability

The original contributions presented in this study are included in the article. Further inquiries can be directed to the corresponding author.
